# Successful management of facial tortuous vascular lesions using long-pulsed 1064 nm Nd:YAG laser: A case report

**DOI:** 10.1016/j.jdcr.2026.01.032

**Published:** 2026-02-02

**Authors:** Luis Leobardo Velázquez-Arenas, Sarahi Garay-Enriquez, Daniela Gómez-Guerra

**Affiliations:** a Department of Dermatology, School of Medicine and Health Sciences at Tecnológico de Monterrey, Mexican Council of Dermatology, Nuevo Leon, Mexico; b School of Medicine and Health Sciences at Tecnológico de Monterrey, Nuevo Leon, Mexico

**Keywords:** dermatology, laser therapy, Nd:YAG, varicose veins, vascular lesions

## Introduction

Over the past decades, significant advancements in laser technology have transformed the management of vascular lesions. These innovations emerged in response to the high rates of scarring and complications associated with early-generation devices, such as the argon laser.^1^^,^^2^ In that context, Drs John Parrish and Rox Anderson revolutionized dermatology and other medical specialties through the introduction of the theory of selective photothermolysis, establishing the scientific basis for targeted laser therapy.^3^ Among the modern laser systems, the long-pulsed 1064 nm Nd:YAG laser stands out for its deeper dermal penetration and superior performance in targeting larger and deeper vascular lesions, offering a well-tolerated therapeutic alternative for challenging vascular presentations.^4^

Varicose veins are among the vascular lesions that may benefit from these technological advancements. They are defined as a dilated and often tortuous vein, most commonly located in the lower extremities. Varicosities involving the facial and cervical regions, however, are extremely rare, with orbital varices representing the most prevalent subtype in this area. Involvement of the facial vein or its direct branches is rarely documented and often presents with thrombosis at the time of diagnosis.^5^ Here, we present a case of tortuous vascular lesion located on the left cheek in a patient with systemic sclerosis, an association not previously reported in the literature.

## Case report

A 61-year-old female with a history of systemic sclerosis and vasculitis presented with multiple tortuous, varicose-like lesions on the left malar region, including 1 with active bleeding that ceased upon directed compression. On examination, the patient exhibited hallmark features of systemic sclerosis, including skin fibrosis and thickening involving the face and distal extremities, accompanied by prominent telangiectasias and Raynaud’s phenomenon.

Contrast-enhanced computed tomography of the neck demonstrated dermal thickening (11 × 5.5 mm) in the malar region, associated with increased density and caliber of superficial vessels.

**Fig 1:**
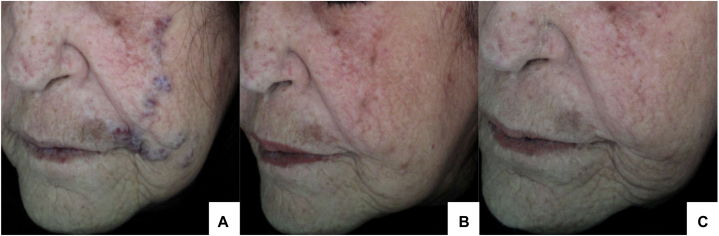
Serial polarized photographs demonstrating progressive resolution of vascular lesions on the left cheek over time. **A,** Baseline, prior to first treatment session (February 28, 2023); **(B)** after the fourth session (July 22, 2023); **(C)** follow-up 2 months after the fifth and final session (February 17, 2024).

The patient underwent treatment with long-pulsed 1064 nm Nd:YAG laser, initiated on February 28, 2023. Simple lidocaine anesthesia was administered prior to each procedure to ensure patient comfort. A total of 5 treatment sessions were performed, delivering 161 cumulative laser pulses, with the last session completed on November 25, 2023 (Table I). Fluence settings were individually adjusted, ranging from 30.4 to 287.8 J/cm^2^, to achieve an optimal clinical response while minimizing the risk of excessive tissue injury and scarring. As a result, multiple treatment sessions were required. The procedure was well-tolerated, and follow-up demonstrated significant reduction in vessel diameter and complete cessation of bleeding episodes, with no evidence of scarring or other complications. The patient remains under ongoing dermatologic follow-up (Fig 1).

**Table I:** Nd:YAG laser treatment parameters by session

Session	Date	Fluence (J/cm2)	Spot size (mm)	Pulse duration (ms)	Number of pulses
1	2/28/2023	43.9	5	55	2
		38.5	5	55	13
		33.1	5	55	2
		140.9	5	55	9
2	3/24/2023	149	5	55	39
		140.9	5	55	31
		157.1	5	55	3
3	4/21/2023	149	5	55	17
4	5/20/2023	165.4	5	55	6
		157.1	5	55	7
		287.8	3	25	5
5	11/25/2023	41.2	5	55	10
		30.4	5	55	3
		140.9	5	55	8
		157.1	5	55	5
		173.7	5	55	1

## Discussion

Several studies have reported positive responses with the use of long-pulsed Nd:YAG laser in the management of various superficial vascular lesions. Beckhor (2006) conducted a study including 35 patients with venous lakes of the lips and cheeks treated with a single session of long-pulsed Nd:YAG laser. Among the 34 patients available for follow-up, 32 achieved complete clearance, while 1 showed a 90% improvement and another a 50% improvement, underscoring its effectiveness and excellent tolerability for facial and labial venous lakes.^6^ Similarly, Ozyurt et al (2012) reported favorable outcomes in a retrospective study of 6 patients with spider angiomas, 130 with facial telangiectasias, and 99 with leg telangiectasias treated with a long-pulsed 1064 nm Nd:YAG laser. Complete or marked improvement was achieved in 100% of spider angiomas, 97% of facial telangiectasias, and 80.8% of leg telangiectasias, confirming the laser’s versatility and tolerability in the treatment of diverse superficial vascular lesions.^7^ Furthermore, John et al (2016) evaluated the use of long-pulsed Nd:YAG laser therapy in 31 consecutive patients with venous lesions of the lip, reporting no evidence of recurrence in 87% of patients at a mean follow-up of 12 months, with only 1 case of a minor contracted scar.^8^ Similarly, Mustafa et al (2023) evaluated the treatment of enlarged veins using a single-session Nd:YAG laser approach. Of the 25 participants included in their cohort, 12 presented with varicose veins located on the cheek. The authors calculated the required laser parameters in advance, determining pulse durations of 15-30 ms and fluences ranging from 110-190 J/cm^2^, with spot diameters between 3 mm and 5 mm based on vessel depth and skin characteristics. This individualized parameter selection resulted in consistently favorable outcomes for cheek lesions.^9^ Collectively, these findings support the long-pulsed Nd:YAG laser as a reliable and valuable therapeutic modality yielding positive responses and durable outcomes in the treatment of vascular lesions involving the face and lips.

## Conflicts of interest

None disclosed.
